# Assessment of humaneness using gunshot targeting the brain and cervical spine for cervid depopulation under field conditions

**DOI:** 10.1371/journal.pone.0213200

**Published:** 2019-02-28

**Authors:** Anthony J. DeNicola, David S. Miller, Vickie L. DeNicola, Robert E. Meyer, Jennifer M. Gambino

**Affiliations:** 1 White Buffalo Inc., Moodus, Connecticut, United States of America; 2 Miller Veterinary Services, PLLC, Loveland, Colorado, United States of America; 3 College of Veterinary Medicine, Mississippi State University, Mississippi, United States of America; University of Mississippi Medical Center, UNITED STATES

## Abstract

Population reduction or eradication of domestic or non-domestic species may be required to address their impacts on the environment, other species, or human interests. Firearms are often used to accomplish these practical management objectives, and there is increased concern that the methods used may compromise animal welfare. We document the accuracy and humaneness of gunshot placement to the brain and cervical vertebrae of Philippine deer (*Rusa marianna*) on Guam during depopulation activities as a model for meeting AVMA standards of euthanasia under field conditions (e.g., animal is not in hand). Deer were shot with a .223 caliber rifle from 10–125 m and approached immediately (<20 s) for assessment. A subset of adult deer was further evaluated for physiological responses including cessation of heart rate, respiration, ocular reflexes, and post-mortem spasms. All deer shot in the brain (n = 132) and upper cervical spine (C1—C3; n = 18) died immediately due to the destruction of the brain or spinal tissue. Shot placements were all within 1.9 cm of the point of aim (i.e., the center of the target region). The accuracy and immediate insensibility resulting from targeting of C1—C3 demonstrates that this is an alternative target site when animal positioning is not optimal for targeting the brain, or there is a need to preserve brain tissue (e.g., Chronic Wasting Disease testing). While targeting of C4 –C7 vertebrae (n = 6) was accurate and resulted in immediate incapacitation, the failure to produce immediate insensibility does not support the use of this shot placement when upper cervical or brain shot placement is an option. It is reasonable to achieve sufficient accuracy to target the brain or upper cervical vertebrae of deer under field conditions and meet standards of euthanasia while accomplishing management objectives.

## Introduction

There is a general societal expectation that the taking of animals’ lives should be conducted as humanely as possible. The American Veterinary Medical Association (AVMA) defines euthanasia as a means of ensuring that animals experience a “good death” [1]. The AVMA also lists a number of factors that should be considered when euthanizing animals, including impacts on the animal, nearby animals, humans, and the environment [1]. For this research, we define euthanasia as a technique that should result in a rapid loss of consciousness followed by cardiac or respiratory arrest and, ultimately, a loss of brain function. However, there are many circumstances during which euthanasia is challenging to achieve, especially during wildlife management activities. Terminating the lives of wildlife under field conditions often presents the challenge of identifying methods that are practical, yet meet societal expectations for humane animal death. When practical limitations that preclude euthanasia exist, those performing the task are still required to strive to achieve as humane a death as possible under the circumstances [2]. Justification for using methods that are not classified as euthanasia (e.g., humane killing—killing performed in a manner that minimizes animal distress, but may not meet the requirements of euthanasia due to situational constraints) may include: circumstances where impacts on humans or the environment (e.g., introduction of environmental contaminants) are judged to be of sufficient magnitude to take precedence over individual animal considerations, where there is a need to avoid adulteration of animals intended for food, or where there are emergency population level concerns such as disease control [1, 3, 4]. Nevertheless, some wildlife population reduction methods are objectionable to some segments of society.

Wildlife professionals often use firearms to terminate animals’ lives during population reduction or disease sampling activities. A corollary to this activity is the perspective that wildlife professionals should receive training and achieve levels of proficiency to ensure that firearm use results in a humane death [5]. This perspective is consistent with societal expectations of competency and interest in animal welfare. Regardless, there is a shortage of evidence-based research demonstrating and supporting the concept that improved firearm practices for wildlife professionals result in better outcomes [5, 6]. Key variables relevant to wildlife professionals’ firearm proficiency include the shooter’s skill [7], equipment used, and judgment during culling activities [8]. In addition, success rates of a given gunshot placement and the resulting impact on humaneness are of interest for evaluating wildlife professionals’ firearm proficiency. While there is research and a theoretical foundation on terminal and wound ballistics that can be used for wildlife management purposes [6], [9–14], further documentation on acceptable euthanasia methods when using firearms is needed to support the interests of wildlife professionals, the animals being impacted, and the public. Specifically, it is important to determine if shot placement to the cervical spine generates adequate hydrostatic shock to render an animal insentient [15] and that the lower brainstem, or phrenic and vagus nerves, are reliably damaged to cease respiration and heart function, resulting in death before returning to consciousness.

Our primary objective was to determine if gunshot placement to the cranium could be reliably accomplished by trained biologists from ≤125 m and would result in euthanasia as defined by the AVMA. Our secondary objective was to determine if gunshot placement to the cervical spine also would result in euthanasia. We hypothesized that upper cervical spine shot placement would result in euthanasia given the anatomy, particularly vital nerve sources and proximity to the medullary and pontine centers, and that lower cervical spine shot placement may result in lack of unconsciousness and prolonged times to death. We assessed lower cervical shot placement given that it is an often-used method by wildlife professionals (thoracic shot placement is also commonly used) and we wanted to demonstrate the relative humaneness. This research will better define the parameters that result in euthanasia when using firearms. Other shot placements may be necessary to meet management objectives and result in a “humane kill.” Regardless of project goals, killing should be accomplished with the most rapid time to unconscious followed by death.

## Study site

Guam is an unincorporated territory of the United States located in the Mariana island chain. The island is the largest in the chain with a land area of 549 km^2^. According to the 2017 census the island has a human population of 164,229. The Navy Base Guam Naval Munitions Site (NBG NMS) is centrally located on the island and contains some of the highest quality remaining native forest and savanna grasslands on Guam. NBG NMS covers a land area of 5,723 ha and provides habitat for 11 Endangered Species Act (ESA)-listed or -candidate species [16]. The NMS is composed of hilly topography with safe earthen backdrops, and facility access by an extensive internal road network with mowed grass areas on each side.

Data collected for this research were part of a greater initiative with the objective of providing a model for an efficient approach that could be applied to future eradication of non-native ungulates on Guam, particularly the Philippine deer. Philippine deer are native to the Philippine Islands [17] and were introduced on Guam in the late 1700s. Deer had become abundant on NBG NMS and were creating biological and safety concerns; especially degrading habitats used by species listed under the ESA [16]. One of the dominant causes of high extinction rates and threats to unique species is the introduction and proliferation of non-native species. Non-native vertebrates, often intentionally introduced by humans, can directly or indirectly lead to species extinction, and land managers globally must address these threats with ambitious eradication efforts [18–21]. Non-native vertebrates are of particular concern on Guam, given the impact of brown tree snakes (*Boiga irregularis*), and the added impact of deforestation caused by non-native ungulates on native species [22]. There were three goals for the overarching project:

1. Intensive population control efforts to reduce overall numbers of non-native ungulates in select areas.
2. Disease surveillance to better understand the risk to humans, domestic animals, and wildlife [23, 24].
3. Provide recommendations and assistance in planning future non-native ungulate control efforts.

## Methods

We collected data as a part of depopulation efforts conducted by White Buffalo Inc. (http://www.whitebuffaloinc.org). The sample sizes were based on what was practical as a part of field operations, where the client had the objective of assessing optimal methods to depopulate non-native ungulates on NBG NMS. Data were collected on four wildlife biologists who received firearm-specific training for remotely euthanizing animals.

### Firearm training

The training included an advanced two-day firearm course that focused on how to apply a firearm as a tool in the wildlife profession (Nuisance Wildlife Control Operator Association- Shooting in Sensitive Environments; NWCOA-SISE). Core content included safety, complete understanding of equipment (including equipment maintenance), proper firearm and ammunition selection, and shot placement to kill efficiently and humanely. Other topics included: 1) shooting skill quantification and development, 2) shooting position assessment and training, 3) development of rapid, short-range, multiple target acquisition shooting skills, 4) shooting from elevated locations (e.g., tree stands and vehicles), 5) night shooting with artificial light, as well as use of thermal and night-vision optics, 6) animal behavior manipulation (e.g., baiting strategies, optimizing engagement advantage, and blending into the environment), and 7) decision-making and situational awareness.

Each firearm training course participant was required to pass a post-training field test to be qualified/certified (NWCOA-SISE Level 2) to humanely kill or euthanize animals under field conditions. Field testing requirements were as follows: consistent demonstration of safe firearm handling and backdrop assessment, conservative decision-making while shooting, full awareness of individual and selected equipment limitations, and demonstration of advanced firearm knowledge and skills for use in complex situations. The shooting test required five shots be completed with a centerfire rifle under 35 s (30 s for semi-automatic firearms) at ranges from 10–100 m with <1.9 cm error from point of aim (POA) (i.e., center of brain). Targets were spaced at 10, 25, 50, 75, and 100 m within a 40 m wide shooting zone. Alternatively, the completion of five shots with a rimfire rifle (or air rifle) under 30 s (25 s for a semi-automatic firearm) at ranges from 10–50 m with <1.9 cm error from POA was also acceptable. Targets were spaced at 10, 20, 30, 40, and 50 m within a 40 m wide shooting zone. All shots taken during testing were required to be of high percentage (>98%) based on demonstrated accuracy during individual course evaluations. A final certification (NWCOA-SISE Level 3) was awarded after completion of an apprenticeship under a Level 3 professional. Successful completion of Level 3 training permitted a graduate to operate independently in developed areas. The NWCOA certification requires renewal every two years to help ensure that shooter’s skills are maintained.

### Field euthanasia

Deer were approached at night using a vehicle and a spotlight and shot at a distance of 10–125 m, using a suppressed, bolt-action .223 caliber scoped rifle (Leupold VX3 4.5 X 14) with a mounted bipod [8]. The vehicle was fitted with a platform to ensure benchrest level shooting precision in all directions (360°). Cartridges were loaded with highly frangible 50 gr projectiles (muzzle energy ~1,500 J) (Hornady V-max, Grand Island, Nebraska). Accuracy and precision of firearms (≤1 cm 5-shot groups) was tested on a firearm range prior to shooting operations, and a rangefinder was used to determine distances during shooting operations. Deer were primarily shot while foraging along roadsides given the limited visibility due to dense forest habitat.

The POA selected for most deer in this study was the cranium (n = 132) because the goal was the destruction of the brain so that an immediate and humane death (i.e., euthanasia) could be ensured. The specific cranial POA chosen varied based on deer’s positioning (see Deer and Pig sections at https://vetmed.iastate.edu/vdpam/about/production-animal-medicine/dairy/dairy-extension/humane-euthanasia/humane-euthanasia/anatomical-landmarks). However, the center of the brain was targeted with a <1.9 cm radius error, as is standard during our wildlife depopulation projects. However, a subset of adult individuals was selected to evaluate the effects of targeting the upper cervical vertebrae (C1—C3, n = 18) and lower cervical vertebrae (C4—C7, n = 6) because conventional depopulation methods may target these shot placement locations. Evaluation of deer shot in the lower cervical vertebrae was ceased when it was clear that these individuals were not experiencing immediate insentience, and exhibited extended periods of consciousness until death. Deer shot in the lower cervical vertebrae (C4—C7) that were still alive upon arrival were not terminated with a second shot, so the time it took for the animals to expire could be documented [14]. These animals died due to blood loss from ballistic tissue trauma proximate to the point of impact (POI).

Each deer in this study (n = 156) was sequentially assigned a unique identification number. A subset of deer were immediately approached (<20 s) after shooting to evaluate several parameters. Evaluation of time to insentience and death were assessed through the following: 1) anatomical impact (site and extent of trauma), 2) POA (cranial versus upper or lower cervical), and 3) response after being shot (time to recumbency). Physiological parameters that were measured included time until cessation of heart rate, respiration, ocular reflexes (palpebral and corneal reflexes), the presence of a glazed eye appearance without tracking, and the cessation of whole body and limb post-mortem spasms [25]. In addition, we recorded cranial bullet entry sites to evaluate shooter accuracy by measuring the distance from the POA to the POI using calipers and distance between the shooter and the deer. Descriptive statistics of field data to characterize time until insentience and death, and the outcome of choosing alternate targets (upper and lower cervical vertebrae) were summarized.

A subset of deer was investigated post-mortem via digital radiography (Sedecal APR-VET, Buffalo Grove, Illinois) to evaluate shooter accuracy and terminal ballistic impacts on select tissue. Individually identified deer in each cohort (brain (n = 2), C1 –C3 (n = 14), and C4 –C7 (n = 6)) were sequentially sampled by removal of the head and neck at approximately the C7—T1 junction for assessment and association with field data. These head/neck samples were sealed in individual, leak-proof bags, using methods to minimize biosecurity risks. These measures included personal protective equipment and treatment of exposed areas with disinfectants. These samples were subsequently transported to the Anderson Air Force Base veterinary clinic, and orthogonal views of tissues adjacent to point of impact (POI) were obtained using post-mortem digital radiography [10]. All deer carcasses and samples were disposed of via deep burial or carcass donation for consumption.

### Statistical analysis

Descriptive statistics were calculated for the time in seconds until cessation of heart rate, respiration, and ocular reflexes, as well as the presence of a glazed eye appearance without tracking, and the cessation of whole body and limb post-mortem spasms. Our data included samples from cranial shots (n = 3), upper cervical spine shots (n = 18) and lower cervical spine shots (n = 6). Data was dropped when NA was listed as the value on an analysis-by-analysis basis.

We analyzed our data using SPSS (version 25.0). Depending upon the data distribution, statistical analysis was performed either with Mann-Whitney U test or Student’s two-sample t-test. Shapiro-Wilk tests were used to assess normality. All tests were two-tailed and significance was determined at the 0.05 level. A Mann-Whitney U test was applied to compare the variable of respiration stop time for upper cervical spine shots and lower cervical spine shots. A Student’s two-sample t-test was applied to compare the variable of cessation of eye reflex for upper cervical spine shots and lower cervical spine shots. Heart rate was not compared between treatments as it is not an indicator of immediate insensibility or death [1] but was recorded to demonstrate persisting heartbeat in animals euthanized by gunshot. Further, body and leg spasms were not compared as they also are not evidence of insentience or immediate death, and only denote damage to the brain or cervical spine. These data were collected to document that involuntary movements often occur when deer are euthanized with a gunshot to the brain or cervical spine.

All research was conducted under contract with the U.S. Navy (Cooperative Agreement N40192-14-2-8000), and all animal collection procedures were reviewed and approved by University of Georgia’s Institutional Animal Care and Use Committee (A2014 09–021).

## Results

The weather conditions were similar on all nights of operations; mostly clear skies with intermittent rain events and temperatures from 25-27° C. Deer were not shot when there was precipitation to minimize impacts on equipment and decrease the risk of complications during shooting.

**Fig 1:**
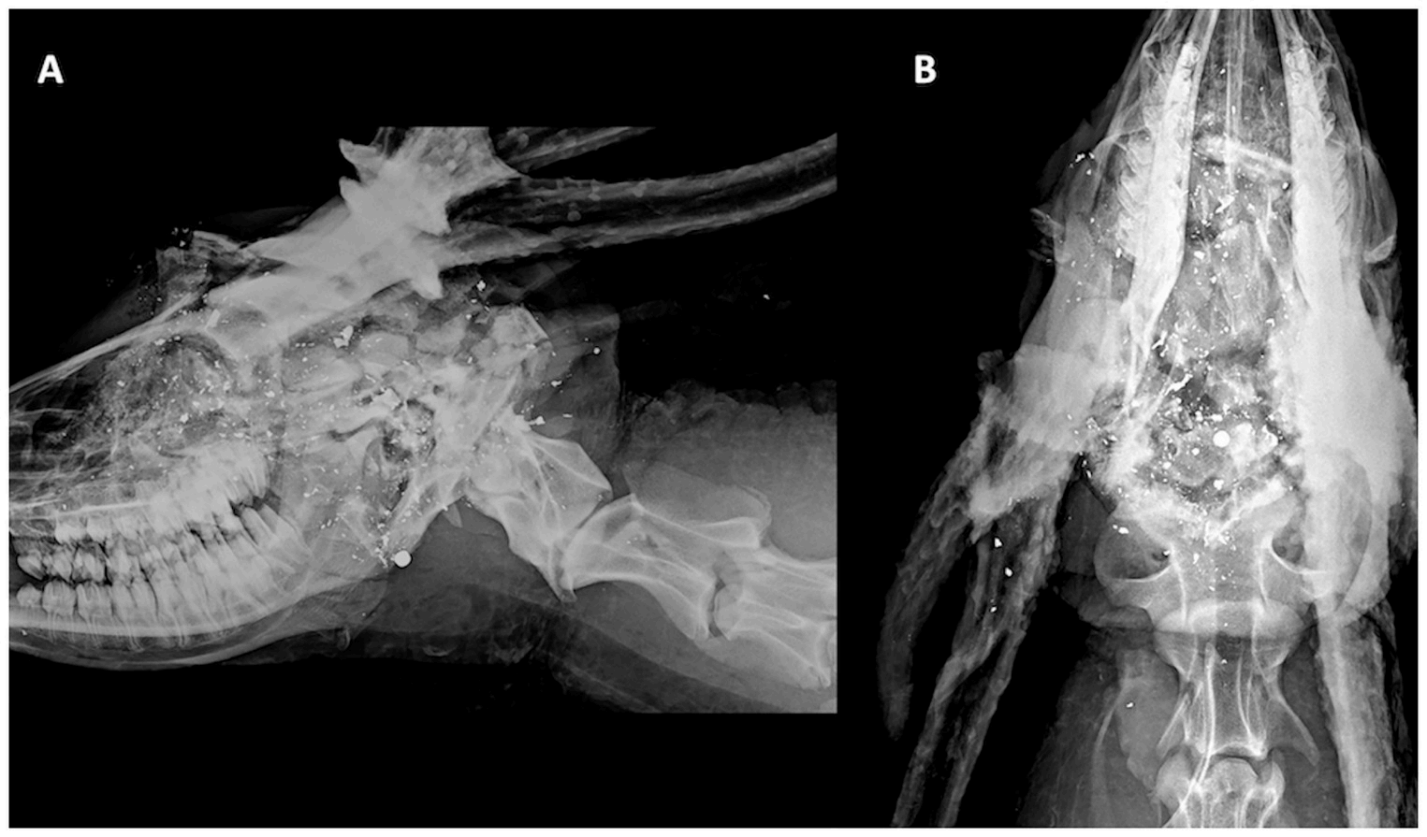
Orthogonal digital radiographs of penetrating ballistic injury in a deer with cranial/brain shot placement. Orthogonal digital radiographs of penetrating ballistic injury in a deer shot with point of aim targeted at the cranium/brain (A) lateral, and (B) ventrodorsal. The caudal skull is the point of impact. There is complete destruction of the osseous calvarium with fragmentation of the dorsal and mid calvarium, frontoparietal region and caudal occiput. Notice the orbital trauma which correlated to the gross finding of globe proptosis. There is widespread traumatic brain injury, collapse of the cranial vault, extensive destruction of the ethmoid labyrinth, herniation of the brain through the cribriform plate and disruption of the hyoid apparatus with pharyngeal swelling and gas tracking caudally through the deep cervical fascial planes. Numerous amorphous, distorted (submillimeter to <2 cm) ballistic lead fragments overlie the resultant multiple skull bone fragments that arise from the skull. The mandibular bone fragments are displaced caudally and temporomandibular joint subluxation. A lesion such as the one described here resulted in instantaneous death.

All deer shot with the cranium (n = 132) as the POA were killed instantaneously, and no follow-up shots were required (Dataset in S1 Dataset). The mean distance from the POI to cranial POAs was 0.73 cm (SE = 0.31, n = 132). Radiographs of a cranially-shot deer are shown in Fig 1. Shot placement to the cranium resulted in the absence of respiration and ocular reflexes in 100% of the animals (Table 1). We did not observe non-spasmodic head and tail movement in any of the cranially shot animals. These animals exhibited glazed eyes and dilated pupils. These observations confirmed death when animals were approached within 20 s from time of shot. However, the heart continued to beat for up to 300 s. Leg paddling and whole-body spasms were observed in most animals shot in the brain (based on general observations), and occurred for up to 290 s, with no gasping or gagging noted (Table 2).

**Table 1:** Mean (SD) time to cessation of physical and physiological parameters following ballistic injury on Philippine deer. Mean (SD) time to cessation in seconds of physical and physiological parameters following ballistic injury associated with three defined shot placements on Philippine deer.

Shot Placement	Cessation of Heartbeat (s)	Cessation of Respiration (s)	Cessation of Eye Reflex (s)^1^	Cessation of Body Spasm (s)	Cessation of Leg Spasm (s)	Eye Tracking^2^
Cranial (n = 3)	275 (35)	0 (0)	0 (0)	97 (167)	0 (0)	0
C1—C3 (n = 18)	346 (46)	0 (0)	160 (90)	71 (99)	126 (130)	0
C4—C7 (n = 6)	364 (93)	116 (90)	212 (79)	45 (110)	172 (97)	5

^1^Eye reflex includes palpebral and corneal.

^2^As measured by presence or absence.

**Table 2:** Select data of adult Philippine deer. Philippine deer physiological responses and cessation time in seconds following being shot in the cranium or cervical spine with a .223 caliber rifle from 1–16 April 2015.

ID	Sex	Shot Placement	Cessation of Heartbeat (s)	Cessation of Respiration (s)	Cessation of Eye Reflex (s)^1^	Cessation of Body Spasms (s)	Cessation of Leg Spasms (s)	Eye Tracking	Agonal gasps	Notes
1	M	Cranial	NA	0	0	0	0	N	N	Control
2	F	Cranial	300	0	0	290	0	N	N	Control—Spasm started at 2:45
3	F	Cranial	250	0	0	0	0	N	N	Control
4	M	C2	NA	0	60	0	0	N	N	
5	M	C1—C2	NA	0	79	0	0	N	N	
6	F	C2—C3	350	0	290	0	0	N	N	
7	F	C1—C2	330	0	120	0	295	N	N	
8	F	C3	390	0	173	0	195	N	N	
9	F	C3	315	0	270	0	210	N	N	
10	F	C3	350	0	140	200	0	N	Y	
11	F	C3	381	0	216	260	190	N	Y	
12	M	C1	352	0	80	170	170	N	Y	
13	F	C3	317	0	200	227	310	N	Y	
14	F	C3	292	0	200	75	0	N	Y	
15	F	C2—C3	321	0	65	140	0	N	N	
16	F	C1—C2	371	NA	NA	NA	NA	N	N	Not located until 1:00
17	F	C3	315	0	230	0	320	N	Y	
18	F	C2—C3	327	0	272	0	205	N	Y	
19	F	C2—C3	290	NA	NA	NA	NA	N	N	Not located until 2:50
20	F	C3	350	NA	NA	NA	NA	N	N	Not located until 4:38
21	F	C1	480	0	0	0	0	N	N	
22	F	C4	364	260	345	270	0	Y	Y	Shallow, erratic breathing
23	F	C4	245	55	231	0	140	Y	Y	Erratic breathing, ear movement
24	F	C4	264	95	110	0	229	Y	Y	Erratic breathing
25	M	C4	450	125	230	0	270	Y	Y	Erratic breathing
26	F	C4	470	0	177	0	230	N	Y	
27	F	C6—C7	390	160	180	0	165	Y	Y	Erratic breathing

^1^Eye reflex includes palpebral and corneal.

**Fig 2:**
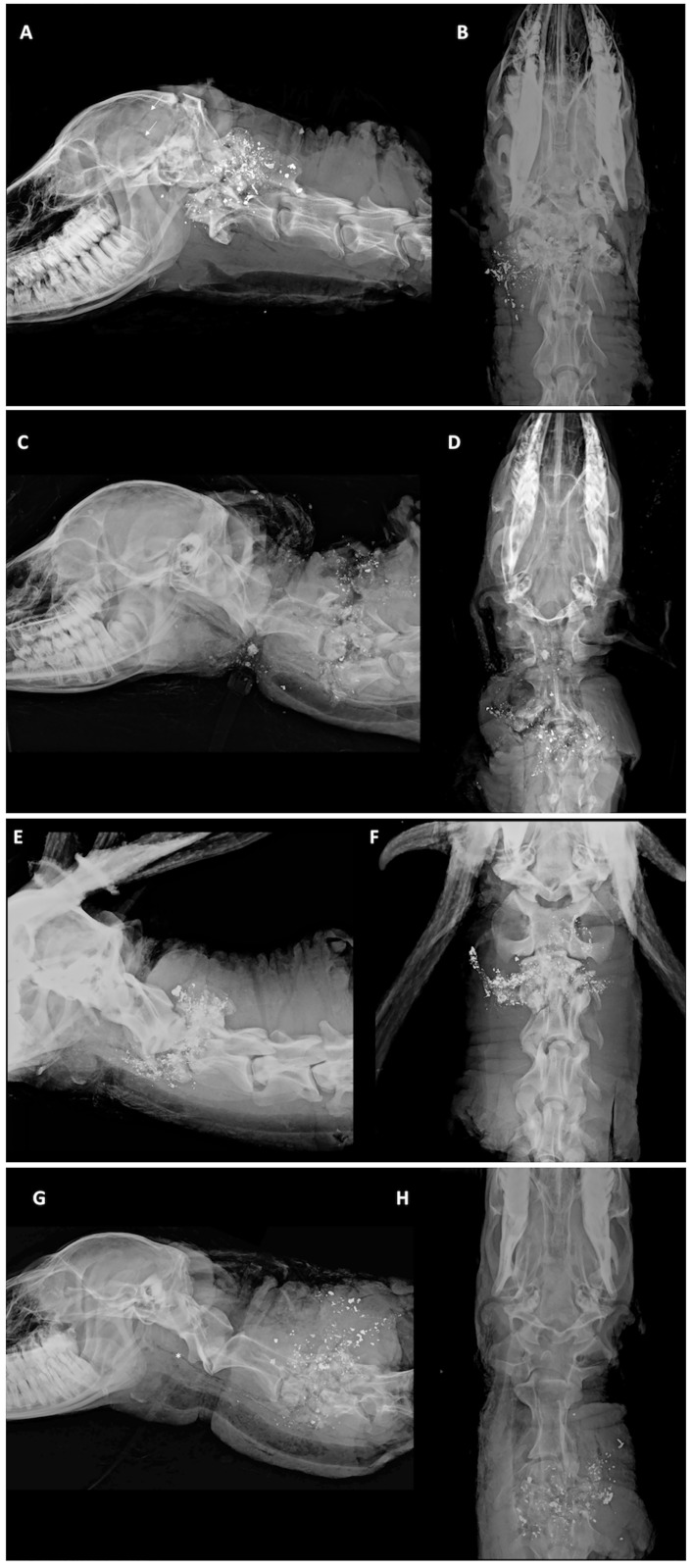
Orthogonal digital radiographs of penetrating ballistic injury in a deer with C1-3 shot placement. Orthogonal digital radiographs of penetrating ballistic injury in a deer shot with the point of aim targeted at C1-3 (A), (C), (E) lateral; and (B), (D), (F) ventrodorsal. The cranial cervical spine is the point of impact. Regional destruction is noted. Numerous amorphous, distorted (submillimeter to <2 cm) ballistic lead fragments overlie the resultant skull and C1-2 bone fragments. Bone fragments have a “pulverized” appearance resulting from combined ballistic kinetic features related to velocity, mass, and surface area, and the rotational forces (or tumbling) imposed on the projectile when it impacts the target. Notice the comminuted, lucent fracture lines (arrows) coursing rostrally through the calvarium and fracture of the occiput, occipital condyle, and petrous temporal bones. This demonstrates the explosive nature of the impact. There is extensive fragmentation and disruption of the normal architecture of C1 and C2, the vertebral spinal canal alignment and the normal anatomic relationships of the atlanto-occipital junction to C2. Numerous amorphous ballistic lead fragments overlie the many bones of the affected cranial cervical spine demonstrating the transfer of kinetic energy to the tissues beyond the path of the projectile and the related collateral tissue damage. There is complete disruption of the spinal canal with compression and collapse of C1-2 and associated soft tissue swelling (edema and hematoma formation). As the point of impact moves caudally (at C2 and C3), the caudal calvarium is preserved with deformation and collapse of the vertebral arch, lamina, and intervertebral disc space at C2-3. Note the bullet fragments in the spinal canal at C2, missile fragmentation and the circumferential destruction and obliteration of the spinal canal. Although not visible, the spinal cord was also obliterated (confirmed on post-mortem evaluation). In other cases of cranial cervical point of impact, traumatic disarticulation (subluxation/luxation) was observed. The soft tissue destruction can extend ventrally to the laryngeal soft tissues resulting in gas tracking through the deep fascial planes of the neck, and pharyngeal and esophageal perforation. In (G) and (H), the gas throughout the length of the esophagus (asterisk) likely resulted from post-mortem tissue handling.

Shot placement to C1 –C3 (n = 18) resulted in the immediate unconsciousness and absence of respiration in all animals, as well as profound terminal tissue impacts that are illustrated in Fig 2. Although most animals were approached within 20 s of the shot impact, many animals were reached within a few seconds, and the results were identical to those assessed after a longer period. The terminal ballistic tissue trauma was similar regardless of the time of approach, so we concluded the results were instantaneous, and not dependent on assessment timelines. Heartbeat continued for 290 s to 480 s. Eye reflexes (palpebral and corneal) persisted in animals for up to 290 s (range: 0–290 s). Mean cessation of ocular reflexes in deer shot in the upper cervical spine did not differ from the mean cessation of ocular reflexes for deer shot in the lower cervical spine shot (two-sample t-test t(19) = -1.3, p = .226). However, no deer were aware of our presence and only demonstrated a blank stare with no eye tracking noted. Some deer shot at, or near C3 (n = 4) exhibited agonal gasping but did not appear to be conscious. There were no body/leg spasms in 5 deer, and in the remaining animals, it persisted for 75 s to 320 s.

**Fig 3:**
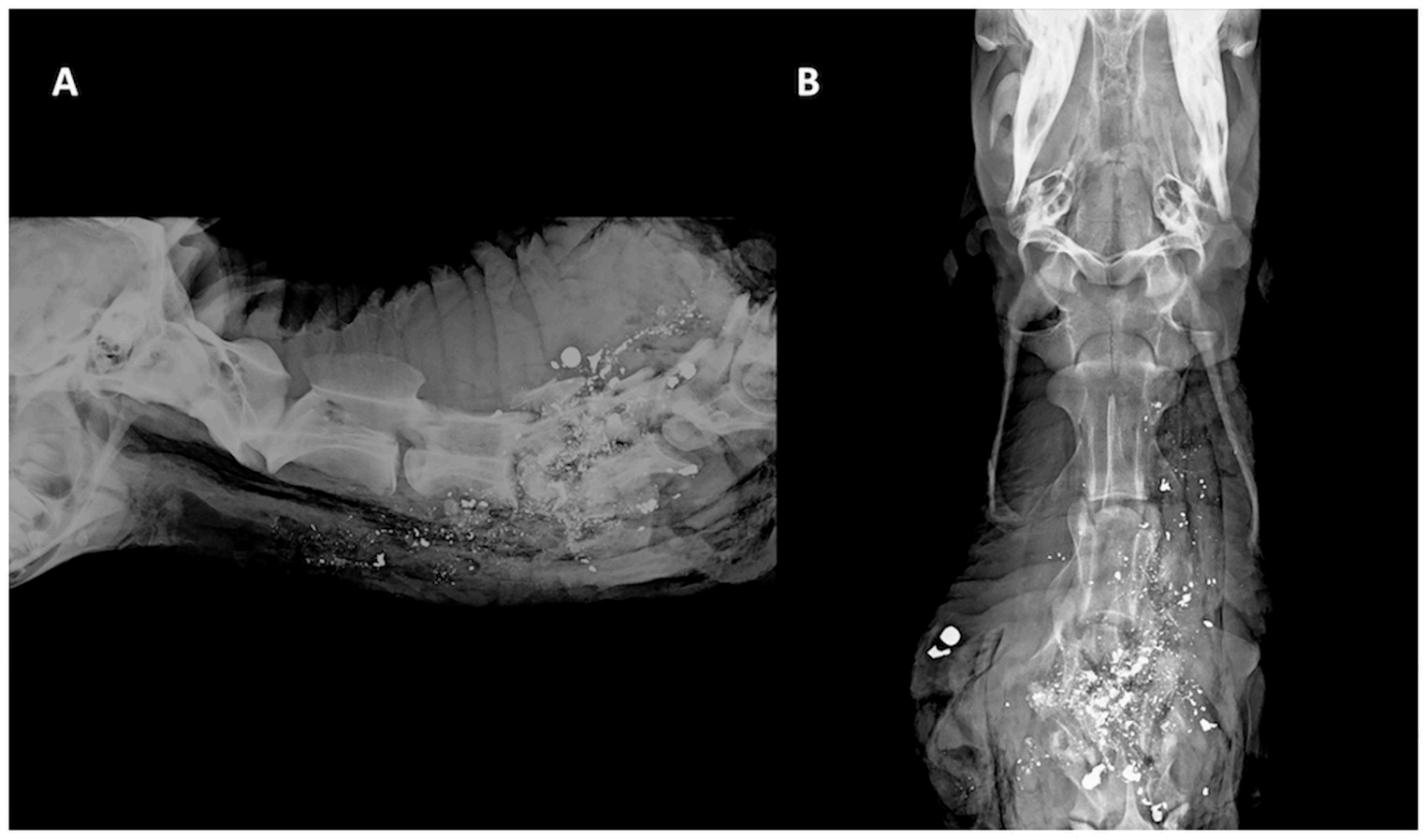
Orthogonal digital radiographs of penetrating ballistic injury in a deer with C4-7 shot placement. Orthogonal digital radiographs of penetrating ballistic injury in a deer shot with C4-7 as the targeted point of aim (A) lateral, and (B) ventrodorsal. The caudal cervical spine is the point of entry. Regional destruction is again noted. There is extensive, comminution of C4, dorsal angulation of the spine, involvement of the articular processes and extensive soft tissue swelling. Metallic projectile fragments are noted within the vertebral canal. When present at the intervertebral disc space, projectile fragments may also be associated with nucleus pulposus extrusion (not shown).

**Fig 4:**
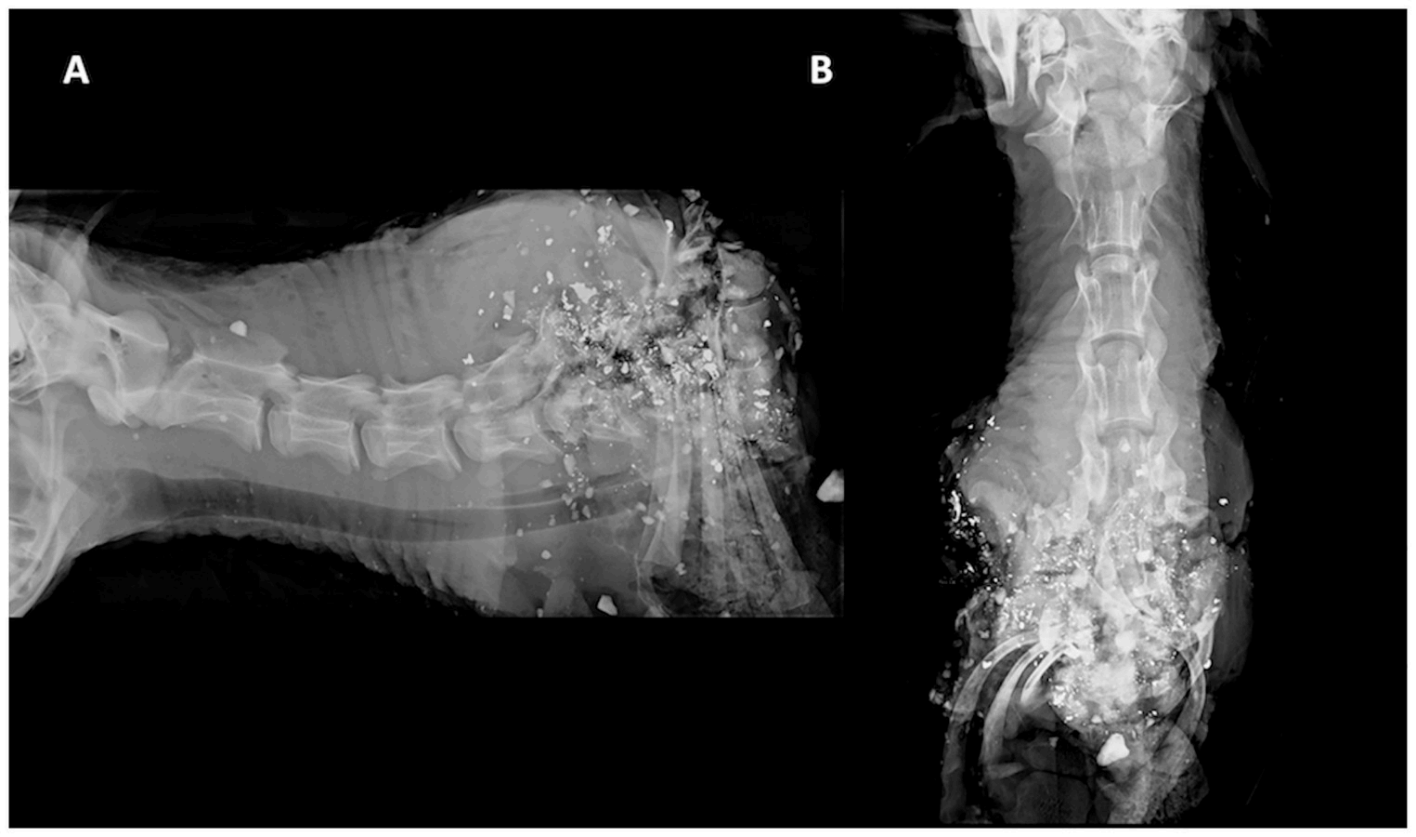
Orthogonal digital radiographs of penetrating ballistic injury in a deer with caudal cervical spine shot placement. Orthogonal digital radiographs of penetrating ballistic injury in a deer shot with the caudal cervical spine as the point of aim (A) lateral, and (B) ventrodorsal. C6-7 is the point of entry. There is circumferential trauma extending from C5-6 to the T3 and ribs. There is extensive disruption of spinal alignment with cranial displacement of the first three ribs and ventrocaudal rotation of the cranial thoracic spine. A complete distortion of the anatomic relationship between the neck and thorax is noted. There is luxation of the C5-6 articular processes and obliteration of the normal contours of C7 and the spinous processes of T1-3. Notice how the skull, cranial cervical spine and disarticulated (but intact) T1-3 vertebral bodies are preserved with the exception of a small chip fracture from the caudodorsal aspect of T1 which is likely secondary to a projectile fragment. Gas within the deep soft tissues of the neck, cranioventral mediastinum and thoracic inlet with a pneumothorax and atelectatic cranial lung field (which is partially attributed to post-mortem tissue handling). Despite the overwhelmingly destructive tissue damage, the injuries and immediate incapacitation associated with lower cervical targeting were sub-lethal, and the target remained conscious and aware. The caudal cervical impact did not result in satisfactory euthanasia.

Shot placement to C4 –C7 (n = 6) resulted in the immediate absence of respiration in only one animal that was shot at the junction of C3/C4, whereas respiration continued for 55 s to 260 s in the other five animals. Respiration stop times for lower cervical spine shots (mean rank = 17.25) were significantly longer than for upper cervical spine shots (mean rank = 8.5) (Mann-Whitney U = 7.5, z = -3.907, p < .0005). No animals were killed instantly. Although all deer were rendered immediately recumbent and incapacitated (limited cervical mobility) with C4—C7 shot placement (Figs 3 and 4), the time until death was protracted for up to 260 s (based on cessation of respiration and eye tracking). Heartbeat continued for 245 s to 470 s. Eye reflexes (blink, palpebral, corneal) persisted in all animals for up to 345 s (range: 110 s– 345 s). Except for the deer shot at the C3/C4 junction, all deer were seemingly aware of our presence (eyes could trace movements) for up to 140 s and had erratic breathing. Agonal gasps were noted in all deer. Body and leg spasms occurred in animals for up to 290 s (range: 140 s– 290 s). The use of the C4 –C7 shot placement was discontinued after six deer were shot, as it became evident that euthanasia (as defined by the AVMA) was not an outcome.

Bullet fragments were limited to the cranium when the cranium was the POA because of the frangibility of the projectiles, (as previously found in [10]). There also were no exiting fragments when the cervical spine was targeted.

## Discussion

This study describes targeting of the cranium for field euthanasia with gunshot of 132 Philippine deer and successful use of this method at distances of 10 m to 125 m under field conditions. The instantaneous destruction of brain tissue in these deer meets euthanasia criteria established by the AVMA [1]. The 100% success rate for cranial POAs indicates that shooters using training methods described and that pass testing requirements have a low risk of inaccurate targeting that could result in wounding and prolonged suffering. Loss of palpebral and corneal reflexes, loss of spontaneous respiration, and severe physical destruction of the cranium indicate that there is extensive damage to the medulla and the reticular formation with cranial shot placements. This tissue damage indicates that deer were immediately rendered unconscious when shot, much as identical observations unequivocally indicate unconsciousness in animals slaughtered in abattoirs [25]. The persistence of a heartbeat and clonic movements also are consistent with cattle slaughter with a penetrating captive bolt [25]. To minimize pre-euthanasia distress, we only engaged deer if there was a high likelihood of being able to cull all animals in the group. This decision was calculated based on group size, distance from protective vegetative cover, and deer temperament in the presence of our vehicle. Deer associated with, or proximate to, deer that were shot did not appear afraid but confused when the first animal was euthanized because they were not familiar with the threat of firearms. This response is very common in a broad spectrum of ungulates that have not been hunted or culled previously. We structure our training and management techniques for precision and speed so that the target animals do not have time to transition from confusion to flight.

Targeting of C1—C3 vertebrae also was accurate and resulted in prompt insensibility and death, thereby providing an alternative target site when animal positioning is not optimal for targeting the cranium, or when there is a need to preserve brain tissue. With the increasing prevalence of Chronic Wasting Disease (CWD), and the need to perform detection sampling or depopulation to reduce disease spread, the value of these data becomes more relevant. To validate the presence of CWD, the obex is often tested if the retropharyngeal lymph nodes do not test negative. Gunshot placement to the cranium, with the select caliber and projectile, rarely impacts the retropharyngeal lymph nodes, but often prevents adequate sampling of the obex. Upper cervical shot placement resulted in the loss of spontaneous respiration, although palpebral and corneal reflexes remained.

In contrast to deer shot in the lower cervical spine (C4 –C7), deer shot in the C1 –C3 region did not exhibit spontaneous eye tracking, thereby indicating that these animals were insentient during the time period prior to cessation of all vital signs [25, 26, 27], and no deer returned to consciousness. It appeared that immediate cessation of respiration and normal cardiac function caused anoxia resulting in eventual loss of ocular reflexes while still insentient. In the interim, dilated pupils and lack of eye tracking indicated a lack of consciousness. The presence of either ocular reflexes or rhythmic breathing does not necessarily indicate consciousness [26, 27]. Our observations on cessation of ocular reflexes in deer shot in the C1-C3 region appeared to be similar to a study on calves bled without stunning, where the corneal reflex was abolished between 55 and 126 s after the EEG indicated unconsciousness [28]. Similar to cranial shot placement, cardiac function was the last measured parameter to be lost, as also was found when euthanizing horses with intrathecal administration of lidocaine or barbiturate overdose applied IV [29, 30].

While targeting of C4—C7 vertebrae was accurate and resulted in immediate incapacitation, the failure to result in immediate deaths does not support the use of this as a humane site for terminating deer lives if upper cervical or brain shot placement is an option. This is especially of concern if an immediate second kill shot is not possible. Shot placement to C4 –C7 resulted in the immediate absence of spontaneous respiration in only one animal, shot at the junction of C3/C4. All deer in this group were aware of our presence (eyes could trace movements) and had erratic breathing and subsequent agonal gasps. There does not appear to be adequate hydrostatic shock when the C4-C7 region is targeted to render an animal unconscious [15], and the phrenic and vagus nerves are not reliably damaged to cause immediate cessation of respiration or interfere with normal heart function. Although some wildlife professionals consider this shot placement to be adequate because it results in immediate incapacitation, we sternly suggest that more humane shot placement is used when it is an option. However, there may be situations where alternative shot placement may be justified based on the circumstance, and "humane killing" is acceptable. For example, deer near airport runways may need to be incapacitated immediately, versus thoracic shot placement where escape behavior is exhibited before death, followed by prompt administration of a cranial shot. Lower cervical shot placement is more reliable at longer distances (e.g., >200 m) when larger caliber firearms are safe to use. Reduced precision at distances greater than were acceptable for this research requires greater terminal energy to increase temporary and permanent wound channel diameter to ensure immediate incapacitation. With increased projectile energy comes the risk of bullet pass through when less tissue mass is present at the POA. Larger caliber firearms, often with more muzzle energy than those used in this study, are not acceptable for cranial and upper cervical spine shot placement on similar body size-species in developed areas where projectile pass through increases safety risks.

Destruction of ≥30% of brain tissue likely results in the immediate death of animals [12]. Targeting vertebrae C1—C3, with adequate terminal energy, also is likely to result in immediate insensibility and death because this is the location of the medullary and pontine centers for respiratory and cardiovascular regulation [15]. Our observations of the absence of respiration, lack of eye reflex responses or glazed eyes with no tracking, dilated pupils, and an absence of non-spasmodic head or tail movement indicate that all animals shot in the brain or upper cervical spine were immediately insentient and appeared to experience prompt deaths. Leg and whole-body spasms were observed in most animals, and it varied considerably whether full body spasms were exhibited or just leg paddling. Body and leg spasms are not always indicative of instantaneous death through the destruction of brain or brain stem tissue and can be associated with damage to the spinal cord [27, 31], as was observed in deer shot in the lower cervical spine. Other vital signs should be monitored to ensure the clonic movements are associated with insentience and subsequent death. The post-mortem movements of deer shot in the cranium and upper cervical spine observed in this study were reflexive movements similar to those observed in cattle rendered unconscious by captive bolt stunning in abattoirs [25]. These post-mortem movements can result in uninformed observers misperceiving that targeted animals are suffering when they are insentient and not capable of feeling pain or experiencing distress. Thus, shooters directing gunshots to the cranium or upper cervical spine must be prepared to explain these post-mortem movements to observers.

Key to this study was training in applied firearm remote euthanasia. Our shot placement success was greater than those documented when night shooting impala, which achieved 93% successful outcomes [32], and similar to sharpshooting urban kangaroos in Australia [33]. It is possible that our success rates were better than documented for impala culling because of our vehicle set up, which allowed for benchrest level stability and precision, combined with greater magnification optics. Training of our shooters was part of an advanced firearm/shooting course for wildlife professionals that was focused on how to apply firearms as a tool for remote euthanasia. Course topics include safety, complete understanding of equipment, proper firearm/ammunition selection, and shot placement to kill efficiently and humanely.

As a consequence of this training, appropriate firearms and ammunition were used in this study to cause a rapid, humane death [11, 12]. We used highly frangible (thin copper jacket and soft lead core) bullets that allowed for rapid expansion and immediate energy transfer to the target tissue. In addition, the following guidelines of operation were used to determine whether a particular shot should be attempted: 1) it was safe to shoot (i.e., there was a safe backstop), 2) euthanasia was judged to be a highly probable outcome, 3) shooting was carried out by experienced, skilled and responsible shooters who had been deemed suitable to the task and had proven marksmanship, 4) deer were within the effective range of the firearm and ammunition being used, and 5) there was sufficient light (night vision/thermal equipment or adequate artificial light) to visualize the optimal POA (the animal could be clearly seen and recognized). Furthermore, shooters minimized psychological stress on deer during depopulation activities by minimizing conspecific awareness of the operation through limited direct line of sight or auditory stimuli. The practicality of this training and predictable outcomes warrants investigation of wider use of these methods in other species.

This study documents that it is reasonable for professional wildlife biologists to achieve sufficient accuracy to target the brain or upper cervical vertebrae of deer under field conditions and thereby meet standards of euthanasia while effectively terminating deer lives. We deliberately avoid use of the term “sharpshooter,” as this may be taken to imply that shooters must reach a level of proficiency that most biologists cannot practically achieve. Counter to this misperception, we have documented that biologists can achieve high levels of accuracy with practical levels of training and practice. Although the four biologists that participated in this study had extensive experience (5 to 21 years using the described methods), most training course participants with basic marksmanship skills can achieve levels of proficiency adequate for some field euthanasia scenarios after completion of the two-day course. For example, we have trained seven US law enforcement agencies and one provincial wildlife agency (48 total participants) to successfully euthanize free-ranging deer using this protocol, with many law enforcement participants being certified only in pistol usage prior to the training. Few participants were unable to establish the necessary skills to humanely and efficiently euthanize deer. All could effectively and reliably euthanize single animals, but a few were unable to apply the methods when multiple animals were present in a dynamic setting. This is important because extensive training and experience are not required before biologists are proficient in the use of gunshot as a means of remote euthanasia for wildlife under many field conditions. However, an apprenticeship under advanced level personnel is advised to accelerate further skill development. An equal level of proficiency is required to target the three anatomical sites used in this study (cranial, upper and lower cervical spine). This level of proficiency is relevant for remote field euthanasia of other species, with firearm and bullet selection accommodation for the relative size of the anatomical features targeted and operator awareness of species-specific anatomy, based on our experience euthanizing other ungulates with firearms. We have experienced the same outcomes for cranial and upper cervical shot placement in feral pigs, feral goats, as well as white-tailed, black-tailed, axis and fallow deer. More research on the specifics of caliber, bullet weight, and muzzle velocity is needed for larger species. Of equal importance is that immediate insentience and rapid death meet the expectations of society at large for a humane death, and this can contribute to acceptance of wildlife management plans that require termination of animals’ lives.

An additional benefit of targeting the cranium and upper cervical vertebrae, versus the lower cervical spine, is that dispersion of metallic fragments from bullets is limited. This appears to present less risk to humans and animals that consume thoracic musculature from these carcasses, compared to lead deposition patterns of hunter-harvested venison where the thoracic cavity is targeted [10, 34]. When CWD sampling using upper cervical shot placement there also is an added benefit of reduced bleeding and tissue dispersion in the field when compared to a cranial POA.

The terminal ballistic properties of the select projectiles significantly decreased pass-through safety risks. The lack of an exit wound with upper cervical shot placement also minimized the shedding of blood and tissue, which is especially important when there is a disease concern. Additional data should be collected for a spectrum of calibers (e.g., .17 HMR, .22 rimfire) and projectiles to evaluate the relative effectiveness and humaneness of lower energy projectiles, particularly for upper cervical shot placement. It is evident, based on the terminal ballistics and effectiveness of the methods used, that there is no value in increasing the size or energy of the projectiles for species of similar stature. This is because larger or more robust projectiles only increase the risk of bullet pass-through and thereby decrease the safety of depopulation programs.

This study’s results should not be interpreted within a limited scope for depopulation of cervids. Training and gunshot accuracy is relevant to all ungulates on the basis of the authors’ experience. Consequently, these methods also are relevant for training emergency responders for disease control depopulation (e.g., CWD) and free-ranging animals affected by severe weather conditions and other natural disasters (e.g., injuries caused by tornadoes or hurricanes, or food shortages due to fire or heavy snowfall), as well as for other purposes. Collection of additional data on other species and under other conditions is ongoing.

## Summary

Our objective was to document that gunshot to the brain of free-ranging deer can be consistently achieved and results in immediate insentience as a means of remote euthanasia. We also assessed the accuracy and outcomes of targeting cervical vertebrae as alternative shot placement options under field conditions that would result in euthanasia without compromising program objectives (e.g., CWD testing of brain tissues). The target site that we evaluated provided useful animal welfare data for management programs where immediate incapacitation is the primary objective for public safety and program efficiency. We documented the ramifications of various shot placements, based on anatomical expectations that would result in incapacitation. This will guide professionals using firearms to kill animals to ensure that they are aware of the relative humaneness of shot placement decisions.

This study documents the success of wildlife biologists trained to accurately shoot deer under field conditions at distances of 10–125 m, as part of a contracted population reduction program for Philippine deer (*Rusa marianna*) in Guam. The training that biologists received and methods employed were practical and reasonably attainable for achieving high levels of gunshot placement accuracy under field conditions. We documented that shots were successfully targeted within a 1.9 cm diameter, that all deer receiving cranium gunshot placement were instantly insentient, and that the methods we used met AVMA standards of euthanasia despite being conducted under field conditions [1, 25]. Sub-studies conducted to evaluate the efficacy of targeting the upper (C1—C3) and lower (C4—C7) cervical vertebrae demonstrated that levels of accuracy matched those for cranial shot placement, but that lower cervical spine targeting did not result in rapid death and did not meet the definition of euthanasia. These data provide a basis for identifying means of improving methods of terminating the lives of free-ranging cervids and the establishment of professional standards. This work also serves as a model for similar remote euthanasia of other wild and domestic ungulates under field conditions. Our ultimate goal is to define humane firearm methods for euthanizing free-ranging animals and develop best management practices for wildlife professionals. Best management practices would acknowledge the methods, equipment, and skill necessary to ensure the most humane death when management projects are implemented.

## Supporting information

S1 Dataset Cranial shot placement physical assessments. (PDF)
