# Supplementary material for: Assessment of humaneness using gunshot targeting the brain and cervical spine for cervid depopulation under field conditions
Source: PLoS One. 2019 Feb 28;14(2):e0213200. doi: 10.1371/journal.pone.0213200 (PMC6395039; doi:10.1371/journal.pone.0213200)
Supplement: S1 Dataset — (PDF) [file pone.0213200.s001.pdf]

Date	Shooter	Sex	Shot Placement	Cessation of Heartbeat (s)	Cessation of Respiration (s)1
4/1/2015	AD	FAWN	cranial	No data	0
4/1/2015	AD	AM	cranial	No data	0
4/1/2015	AD	AF	cranial	No data	0
4/1/2015	AD	FAWN	cranial	No data	0
4/1/2015	AD	AF	cranial	No data	0
4/1/2015	AD	AF	cranial	No data	0
4/1/2015	AD	AF	cranial	No data	0
4/1/2015	AD	FAWN	cranial	No data	0
4/1/2015	AD	AM	cranial	No data	0
4/1/2015	AD	AF	cranial	No data	0
4/1/2015	AD	AF	cranial	No data	0
4/1/2015	AD	FAWN	cranial	No data	0
4/1/2015	AD	FAWN	cranial	No data	0
4/1/2015	AD	AF	cranial	No data	0
4/1/2015	AD	AF	cranial	No data	0
4/1/2015	AD	AF	cranial	No data	0
4/1/2015	AD	AF	cranial	No data	0
4/1/2015	AD	FAWN	cranial	No data	0
4/2/2015	DW	AF	cranial	No data	0
4/2/2015	DW	AF	cranial	No data	0
4/2/2015	DW	FAWN	cranial	No data	0
4/2/2015	DW	AF	cranial	No data	0
4/2/2015	DW	AF	cranial	No data	0
4/2/2015	DW	AM	cranial	No data	0
4/2/2015	DW	AM	cranial	No data	0
4/2/2015	DW	FAWN	cranial	No data	0
4/2/2015	DW	AF	cranial	No data	0
4/2/2015	DW	AF	cranial	No data	0
4/2/2015	DW	FAWN	cranial	No data	0
4/2/2015	DW	AF	cranial	No data	0
4/2/2015	DW	FAWN	cranial	No data	0
4/2/2015	DW	AF	cranial	No data	0
4/2/2015	DW	AF	cranial	No data	0
4/2/2015	DW	AF	cranial	No data	0
4/2/2015	DW	AF	cranial	No data	0
4/2/2015	DW	AM	cranial	No data	0
4/2/2015	DW	FAWN	cranial	No data	0
4/2/2015	AD	AM	cranial	No data	0
4/2/2015	DW	AM	cranial	No data	0
4/2/2015	DW	AF	cranial	No data	0
4/2/2015	DW	FAWN	cranial	No data	0
4/2/2015	DW	AM	cranial	No data	0
4/8/2015	DW	AF	cranial	No data	0
4/8/2015	DW	FAWN	cranial	No data	0
4/8/2015	DW	AF	cranial	No data	0

4/8/2015	JH	AF	cranial	No data	0
4/8/2015	JH	AF	cranial	No data	0
4/8/2015	JH	FAWN	cranial	No data	0
4/8/2015	JH	AF	cranial	No data	0
4/8/2015	JH	AM	cranial	No data	0
4/8/2015	JH	AM	cranial	No data	0
4/8/2015	JH	FAWN	cranial	No data	0
4/8/2015	JH	AF	cranial	No data	0
4/8/2015	JH	FAWN	cranial	No data	0
4/8/2015	JH	AM	cranial	No data	0
4/8/2015	AD	AF	cranial	300	0
4/8/2015	AD	AF	cranial	250	0
4/8/2015	JH	AF	cranial	No data	0
4/8/2015	JH	AM	cranial	No data	0
4/8/2015	JH	AF	cranial	No data	0
4/8/2015	JH	FAWN	cranial	No data	0
4/8/2015	JH	AF	cranial	No data	0
4/8/2015	JH	AM	cranial	No data	0
4/8/2015	JH	AM	cranial	No data	0
4/8/2015	JH	AF	cranial	No data	0
4/8/2015	JH	FAWN	cranial	No data	0
4/8/2015	JH	AF	cranial	No data	0
4/8/2015	JH	AF	cranial	No data	0
4/14/2015	RR	FAWN	cranial	No data	0
4/14/2015	RR	AM	cranial	No data	0
4/14/2015	RR	FAWN	cranial	No data	0
4/14/2015	RR	AF	cranial	No data	0
4/14/2015	RR	AF	cranial	No data	0
4/14/2015	RR	AF	cranial	No data	0
4/14/2015	RR	AF	cranial	No data	0
4/14/2015	RR	FAWN	cranial	No data	0
4/14/2015	RR	FAWN	cranial	No data	0
4/14/2015	RR	AF	cranial	No data	0
4/14/2015	RR	AM	cranial	No data	0
4/14/2015	RR	AF	cranial	No data	0
4/14/2015	RR	AM	cranial	No data	0
4/14/2015	RR	FAWN	cranial	No data	0
4/14/2015	RR	AF	cranial	No data	0
4/14/2015	RR	FAWN	cranial	No data	0
4/14/2015	RR	AM	cranial	No data	0
4/14/2015	RR	AF	cranial	No data	0
4/14/2015	RR	AF	cranial	No data	0
4/14/2015	RR	AM	cranial	No data	0
4/14/2015	RR	FAWN	cranial	No data	0
4/14/2015	RR	AF	cranial	No data	0
4/14/2015	RR	AF	cranial	No data	0
4/14/2015	RR	AF	cranial	No data	0
4/15/2015	AD	AF	cranial	No data	0
4/15/2015	AD	AM	cranial	No data	0
4/15/2015	AD	AF	cranial	No data	0
4/15/2015	AD	AM	cranial	No data	0
4/15/2015	AD	FAWN	cranial	No data	0

4/15/2015	AD	AF	cranial	No data	0
4/15/2015	AD	AF	cranial	No data	0
4/15/2015	AD	AM	cranial	No data	0
4/15/2015	AD	AF	cranial	No data	0
4/15/2015	AD	AF	cranial	No data	0
4/15/2015	AD	AF	cranial	No data	0
4/15/2015	AD	AF	cranial	No data	0
4/15/2015	AD	AF	cranial	No data	0
4/15/2015	AD	FAWN	cranial	No data	0
4/15/2015	AD	AF	cranial	No data	0
4/16/2015	AD	AF	cranial	No data	0
4/16/2015	AD	AM	cranial	No data	0
4/16/2015	AD	AM	cranial	No data	0
4/16/2015	AD	AM	cranial	No data	0
4/16/2015	DW	FAWN	cranial	No data	0
4/16/2015	DW	FAWN	cranial	No data	0
4/16/2015	DW	FAWN	cranial	No data	0
4/16/2015	DW	AF	cranial	No data	0
4/16/2015	DW	FAWN	cranial	No data	0
4/16/2015	DW	AM	cranial	No data	0
4/16/2015	DW	AF	cranial	No data	0
4/16/2015	DW	FAWN	cranial	No data	0
4/16/2015	DW	AF	cranial	No data	0
4/16/2015	DW	AF	cranial	No data	0
4/16/2015	DW	AF	cranial	No data	0
4/16/2015	DW	AF	cranial	No data	0
4/16/2015	DW	AF	cranial	No data	0
4/16/2015	DW	AF	cranial	No data	0
4/16/2015	DW	AF	cranial	No data	0
4/16/2015	DW	FAWN	cranial	No data	0
4/16/2015	DW	FAWN	cranial	No data	0
4/16/2015	DW	FAWN	cranial	No data	0
4/16/2015	DW	FAWN	cranial	No data	0
4/16/2015	DW	AF	cranial	No data	0
4/16/2015	DW	FAWN	cranial	No data	0

1 - upon arrival to carcass
2 - upon arrive to carcass
3 - measured to the nearest 0.5 cm

Cessation of Eye Reflex (s)2	Cessation of Body Spasms (s)	Cessation of Leg Spasms (s)	Time to recumbancy (s)	Image #	POI to POA (cm)3	Distance to Animal (m)
0	No data	No data	0	None	0.3	14
0	No data	No data	0	None	1	40
0	No data	No data	0	None	0.9	33
0	No data	No data	0	None	0.2	79
0	No data	No data	0	None	0	23
0	No data	No data	0	None	1.1	47
0	No data	No data	0	None	1.3	43
0	No data	No data	0	None	0.6	92
0	No data	No data	0	None	0.5	55
0	No data	No data	0	None	1.4	107
0	No data	No data	0	None	0.3	12
0	No data	No data	0	None	1.2	67
0	No data	No data	0	None	0	36
0	No data	No data	0	None	0.6	87
0	No data	No data	0	None	1.7	21
0	No data	No data	0	None	0.4	25
0	No data	No data	0	None	1.3	61
0	No data	No data	0	None	1.8	97
0	No data	No data	0	None	1.3	112
0	No data	No data	0	None	1.2	32
0	No data	No data	0	None	0.7	35
0	No data	No data	0	None	1	77
0	No data	No data	0	None	1.5	49
0	No data	No data	0	None	0.3	11
0	No data	No data	0	None	1.6	24
0	No data	No data	0	None	1	89
0	No data	No data	0	None	1.2	93
0	No data	No data	0	None	0	56
0	No data	No data	0	None	0.5	12
0	No data	No data	0	None	1.1	122
0	No data	No data	0	None	1.3	16
0	No data	No data	0	None	1.7	21
0	No data	No data	0	None	0.4	19
0	No data	No data	0	None	0.8	64
0	No data	No data	0	None	1	99
0	No data	No data	0	None	1.2	111
0	No data	No data	0	None	0.2	32
0	0	0	0	AK22	0.6	68
0	No data	No data	0	None	0.8	74
0	No data	No data	0	None	0.7	57
0	No data	No data	0	None	1.2	60
0	No data	No data	0	None	0.2	79
0	No data	No data	0	None	0.3	31
0	No data	No data	0	None	0	91
0	No data	No data	0	None	1.4	44

0	No data	No data	0	None	0.9	103
0	No data	No data	0	None	0.3	10
0	No data	No data	0	None	0.7	19
0	No data	No data	0	None	0	20
0	No data	No data	0	None	1.6	111
0	No data	No data	0	None	0.2	26
0	No data	No data	0	None	1.1	36
0	No data	No data	0	None	0.2	40
0	No data	No data	0	None	0	15
0	No data	No data	0	None	1.5	94
0	290	0	0	None	0	30
0	0	0	0	NK4	0.6	56
0	No data	No data	0	None	1	106
0	No data	No data	0	None	1.2	73
0	No data	No data	0	None	0	21
0	No data	No data	0	None	0.6	26
0	No data	No data	0	None	1.2	76
0	No data	No data	0	None	1.3	43
0	No data	No data	0	None	0	38
0	No data	No data	0	None	0.5	49
0	No data	No data	0	None	1.7	63
0	No data	No data	0	None	1.6	96
0	No data	No data	0	None	1.2	39
0	No data	No data	0	None	1	50
0	No data	No data	0	None	1.4	72
0	No data	No data	0	None	0	66
0	No data	No data	0	None	0.7	104
0	No data	No data	0	None	0.3	29
0	No data	No data	0	None	1.8	88
0	No data	No data	0	None	0	44
0	No data	No data	0	None	1.3	47
0	No data	No data	0	None	0.2	34
0	No data	No data	0	None	0	80
0	No data	No data	0	None	0.4	47
0	No data	No data	0	None	0.7	61
0	No data	No data	0	None	1.7	118
0	No data	No data	0	None	0.3	16
0	No data	No data	0	None	0	23
0	No data	No data	0	None	1.2	57
0	No data	No data	0	None	0	51
0	No data	No data	0	None	0	24
0	No data	No data	0	None	0.6	75
0	No data	No data	0	None	0.3	30
0	No data	No data	0	None	1	23
0	No data	No data	0	None	0.2	64
0	No data	No data	0	None	0.7	29
0	No data	No data	0	None	0.4	81
0	No data	No data	0	None	1.1	107
0	No data	No data	0	None	0	46
0	No data	No data	0	None	0.5	39
0	No data	No data	0	None	0	53
0	No data	No data	0	None	0.3	11

0	No data	No data	0	None	0.8	88
0	No data	No data	0	None	1.4	89
0	No data	No data	0	None	0	74
0	No data	No data	0	None	0.4	90
0	No data	No data	0	None	0	33
0	No data	No data	0	None	0.6	54
0	No data	No data	0	None	0.3	16
0	No data	No data	0	None	0.9	19
0	No data	No data	0	None	0.2	13
0	No data	No data	0	None	1	69
0	No data	No data	0	None	0	45
0	No data	No data	0	None	0.4	21
0	No data	No data	0	None	1.8	90
0	No data	No data	0	None	0.3	14
0	No data	No data	0	None	1.5	32
0	No data	No data	0	None	0	80
0	No data	No data	0	None	1.4	67
0	No data	No data	0	None	0.9	41
0	No data	No data	0	None	0.6	36
0	No data	No data	0	None	1	83
0	No data	No data	0	None	0	50
0	No data	No data	0	None	0.7	42
0	No data	No data	0	None	1.1	37
0	No data	No data	0	None	1.6	115
0	No data	No data	0	None	0.3	76
0	No data	No data	0	None	0	49
0	No data	No data	0	None	1.6	93
0	No data	No data	0	None	0.8	85
0	No data	No data	0	None	0.7	66
0	No data	No data	0	None	0.3	61
0	No data	No data	0	None	1.4	14
0	No data	No data	0	None	0	33
0	No data	No data	0	None	0.5	20
0	No data	No data	0	None	1.4	101
0	No data	No data	0	None	1.2	74
